# The Influence of A Cross-Channel Extrusion Process on The Microstructure and Properties of Copper

**DOI:** 10.3390/ma12233995

**Published:** 2019-12-02

**Authors:** Radosław Łyszkowski, Magdalena Łazińska, Dariusz Zasada

**Affiliations:** Faculty of Advanced Technology and Chemistry, Military University of Technology, 00-908 Warsaw, Poland; magdalena.lazinska@wat.edu.pl (M.Ł.); dariusz.zasada@wat.edu.pl (D.Z.)

**Keywords:** severe plastic deformation (SPD), cross-channel extrusion (CCE), back pressure (BP), structure, resistivity

## Abstract

A new cross-channel extrusion (CCE) method with the application of a back pressure (BP) is proposed and experimentally tested. The introduction of pressure blocks the free flow of material by using an additional set of pistons, which prevents the loss of consistency. The paper presents results of experimental trials of CCE process. Between one and eight passes of CCE with and without a BP were applied to pure copper billets to refine their initial coarse-grained microstructure at room temperature. It was found that processing by CCE results in the formation of a lamellar structure along the extruded axis and the fine-grained structure in the remaining volume. The material exhibited dynamic recrystallization, which results in the formation of 0.5- to 2-μm grains after one pass and 2- to 8-μm grains after four CCE passes. The fine-grained material had YS of 390-415 MPa. An increase in the microhardness from 70 to 130 HV02 after one pass and then a decrease after four passes were observed. This might indicate that secondary recrystallization and selective grain growth occur, because an exothermic peak (158.5 °C, 53 ± 2.1 J/mol) was observed during DSC (differential scanning calorimetry) testing. The resistivity of the once deformed copper significantly decreases, while its further processing causes the resistivity to increase.

## 1. Introduction

In conventional plastic forming processes, such as compression or rolling processes, the thickness/amount of material in the plane of deformation limits the maximum deformation. An equally important limitation is the material’s consistency without any decohesion effects. This is especially important for materials with limited plasticity and susceptibility to applied treatments. The machining process carried out with the use of severe plastic deformation (SPD), due to the more complex state of stress, which allows for much larger deformation [1,2,3,4]. The underlying shear stress system and the change in the deformation route are very effective methods of plastic processing, and these techniques are currently regarded as one of the most effective ultrafine-grain (UFG) methods for metals and alloys [5,6]. The original grain structure modifies its shape and size during large plastic deformation. UFG materials are characterized by very high strength when compared to their coarse-grained counterpart [7,8,9]. In these methods, the amount of deformation usually exceeds the values that were obtained by classical methods, but their effective implementation requires the precise selection of deformation conditions and the use of special devices and tools [10,11,12].

**Figure 1:**
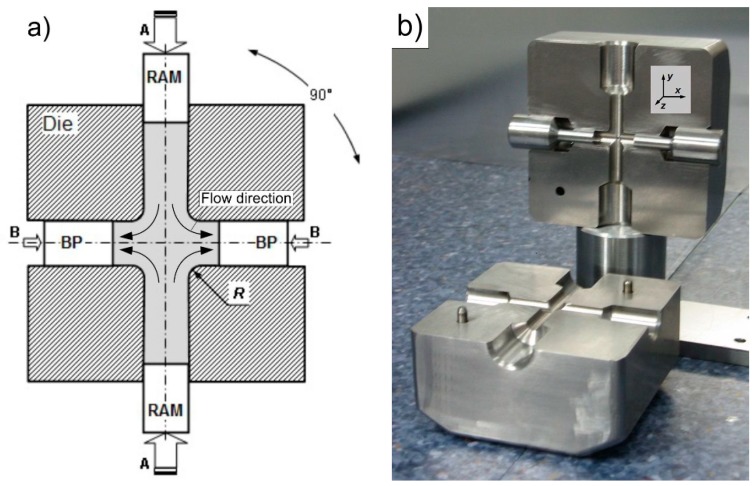
Of cross-channel extrusion with back pressure (**a**) and view of die (**b**).

In recent years, SPD-techniques have attracted increasing attention due to their potential for the fabrication of bulk nanostructures and UFG materials [13,14]. Currently, high pressure torsion (HPT) [15], equal channel angular pressing (ECAP) [16], and accumulative roll bonding (ARB) are the most common SPD methods [17,18]. Cross-channel extrusion (CCE), which is also called cross-equal channel angular pressing (C-ECAP), is a relatively new method that is a type of SPD technique. The solution is based on the use of a cross matrix channel system (Figure 1a), in which the material is compressed in two opposite directions (pistons A), which thus causes the system to reorganize and outflow in two opposite directions that are perpendicular to the original directions [19,20]. The use of a second set of pistons (B) ensures the possibility of blocking the free flow of material in the lateral direction (back pressure BP), which promotes the creation of favorable compressive stresses [21,22,23].

This method allows for a large accumulated strain as a result of the basic extrusion process repetition, in which the die is rotated 90° without removing material from the inside of the die. However, the implementation of this method is difficult and threatened by the appearance of cracks due to the complex state of the stress and the possibility of using processing materials with a limited plasticity [24]. Hence, inhibiting the outflow of material (pistons B) leads to an increase in the level and range of hydrostatic stresses, thus enabling the production of a material with the required characteristics. The intention is to enable the processing of materials with limited plasticity or being formed by powder metallurgy techniques.

**Figure 2:**
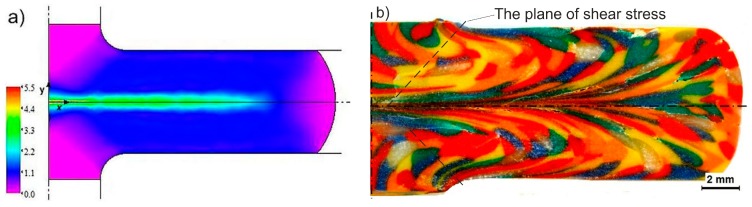
(**a**) And physical simulation (**b**) of deformation after one pass by cross-channel extrusion (CCE) [25].

From previous research, the authors of an article conducted an extensive FEM (finite element) numerical analysis of the CCE process [25]. This study showed that, in the central zone (located at the intersection of both channels), there are very strong three-axis compressive stresses, and the material, along with the movement to the transverse (outlet) channels, undergo shear. This leads to the formation of a relatively homogeneous deformed outer layer on the sample (ε ≈ 1) and a strongly deformed (ε = 3–5) paraxial zone (Figure 2a). These values are similar to those that Ghazani and Eghbali [26] presented. As a result of the accumulation of deformation, the deformation can reach a value of ε ≥ 10 in the paraxial zone and ε = 4 for the remaining area after the 4th cycle. The use of back pressure does not affect the degree of deformation, but it increases its homogeneity and prevents the occurrence of cracks. The extrusion of a clay sample verified the FEM results (Figure 2b). The geometric character of the CCE die as well as the compressive and shearing stress has governed the designed macrostructures. The creation of the highly flattened and elongated grains was observed in the extrusion directions. Based on the obtained information, a device for processing materials while using the CCE method was developed and constructed; the main element of this method is the matrix that is shown in Figure 1b. Pressing the Fe-22Al-5Cr alloy (1–2 passes) verified its operation [25].

Previous deformation studies that were performed while using the CCE method focused on the theoretical analysis of this issue [26,27], and only Chou and colleagues carried out practical tests [19,20]. Chou et al. extruded Sn samples at room temperature and the Al-Mg-Si alloy system at elevated (hot) temperatures. However, this work mainly focused on analyzing the received structures. Therefore, understanding the phenomena occurring during the processing of materials by the CCE method requires further analysis and, in particular, linking the structural changes with the properties (e.g., the mechanical and electrical properties) of the materials tested was evaluated.

Quite a few papers have reported on the impact of SPD processing on the electrical properties of Cu or Al alloys [28,29,30]. Electrolytic copper is used for electrical installations and equipment due to its low electrical resistivity. In addition to high conductivity, the high strength parameters are required for such applications. It is known that mechanical properties can be improved by cold forming metals. Refining the microstructure and increasing the number of grain boundaries inhibit the movement of dislocations and lead to hardening. On the other hand, these changes may have an adverse effect on the metal’s electrical properties. However, the impact of GBs or dislocations on electron scattering is smaller than in the case of other structural defects.

As previously mentioned, a new solution to the problem of processing materials with a limited susceptibility to deformation while using CCE processing has been proposed. This method is based on the concept of CCE with back pressure, which was described and modeled while using a finite element (FEM) simulation [25]. This paper focuses on verifying the feasibility of this idea by conducting a laboratory experiment and investigating the changes in the properties and microstructure of the processed copper billets. This study is very important, especially as deformation increases up to high strain levels. Analyzing the correlation between the microstructure and the mechanical and electrical properties of the deformed sample is of particular interest. Such information is of great relevance to the practical applications of SPD-processed materials.

## 2. Experimental

Figure 1b shows the tool assembly used for the CCE process, and its detailed description can be found in [25]. The assembly consists of two steel blocks that both have cylindrical cross-shaped channels with dimensions of ∅8 mm and a length of 50 mm. An arc with a 2-mm radius connects the channels. Four cylinder punches are used to press the material and match the four channels at each side of the die.

Extrusion started after the work pieces were put in the die. The sample was placed centrally in the vertical channel, the upper and bottom pistons were inserted, and the sample was then extruded. As the process proceeds, the material underwent reorganization and then flowed in opposite directions through the horizontal channel. Compression continues until the moment when the two punches abut on the resting surfaces. This corresponds to an 8 mm clearance between their end faces, which is the channel diameter. The above mentioned process can be defined as a one pass deformation. Subsequently, depending on the required number of passes, the die was rotated by 90°, always in the same direction. A second set of punches was inserted into the die when the sample was deformed with back pressure. Thus, after the rotation of the die, the punch pressing was blocked and the whole extrusion process started again. The work pieces were only removed from the die after the completion of the multi-extrusion process. The specimens were extruded with one to eight passes while using a 1 MN hydraulic press and a ram speed of 2 mm/s at room temperature. Dry graphite lubricant spray was used to reduce the friction at the metal-tool interface.

Commercial copper (purity 99.9%) was machined from a rolled bar with a 10 mm diameter in a stamens with dimensions of ∅8 × 40 mm. The specimen was annealed at 500 °C for one hour in a vacuum to eliminate the hardness and residual stress from the previous metalworking processes and to achieve a recrystallized material with good workability.

The structural characterization of the Cu specimens was carried out by light optical microscopy Nikon MA 200 (Irvine, CA, USA) and FEI Quanta three-dimensional (3D) field emission gun scanning electron microscope (FEG-SEM) (Hillsboro, OR, USA) to analyze the microstructure, grain boundary character distribution (EBSD), and lattice strain in the annealed and extruded material. All of the samples were subjected to metallographic preparation (grinding with up to 4000 SiC papers, diamond polishing, and preparing #0.06 μm silica suspensions) to minimize the negative surface effects on the obtained electron diffraction data. All of the acquired EBSD data sets were “cleaned-up” (i.e., data analysis did not include points with a neighbor confidence index (CI) value below 0.2). The analysis was undertaken on the extruded samples and the vertical-longitudinal and transverse or horizontal-longitudinal cross sections. The measuring area was about 22,500 μm^2^ with a step of 0.8 mm, and the area was analyzed while using TSL OIM Analysis software (v. 5).

A grain boundary character distribution was examined while determining the misorientation angles; angles between 2–15° were assumed to be low-angle grain boundaries (LABs) and angles greater than 15° were high-angle boundaries (HABs). Studying the EBSD data by a local misorientation approach to distinguish the deformed and recrystallized grains [31] and, thus, obtain quantitative information regarding the recrystallized fraction was completed to assess the lattice strain evolution. References [32,33] show the principles of this method.

The amount of stored deformation energy was measured by differential scanning calorimetry (DSC) with a SETARAM Sensys Evo 3D (Lyon, France) apparatus that was equipped with a Calvet-type three dimensional sensor, which allows for accurate measurements of the heat generated, regardless of the shape of the sample [34]. Specimens with dimensions of ∅5 × 2 mm and a mass of approx. of 450 mg were cut out from deformed samples, ground, and then polished. Samples were heated from 20 to 300 °C with a rate of 10 °C/min. The effects of releasing the stored energy were observed during the first run. After reaching 300 °C, the sample was cooled to room temperature and the cycle was repeated. The repeat run second run was used as a blank test in analyzing the results.

The impact of the fragmentation of the material structure on its electrical properties was evaluated by measuring its resistivity. For this purpose, four-wire sensing (4T), which is an electrical impedance measuring technique also known as Kelvin sensing, was used [35]. This method uses two pairs of current-carrying and voltage-sensing electrodes to obtain measurements that are more accurate than those that were obtained by simpler two-terminal (2T) sensing, thus eliminating the lead and contact resistance from the measurement. This is a benefit in the accurate measurement of small resistances. A pair of current leads powers the circuit. There is a voltage drop on the measured element, according to Ohm’s law *U* = *IR*. Supplying voltage leads directly to the sample does not cause a voltage drop in the power cables, which is negligible.

For the resistance measurements, three copper samples were prepared in the initial state (homogenized) after one and four cycles of CCE and four cycles of CCE with additional heating at 200 °C for 10 min. Afterwards, 3 × 2 mm plates (EDM cut from the mid-section, X-Y) were mechanically ground to a thickness less than 0.2 mm. The measurement was made with a CEM DT.5302 milliohmmeter (Shenzhen, China) in the range of 0–400 mΩ while considering the short circuit resistance (0.6 mΩ) of the device. The measured resistance was related to the dimensions of the sample, thus determining its resistivity [36].

A tensile test and micro-hardness measurements were carried out to validate the effect of the structural evolution of the investigated material. The mechanical properties of the samples were evaluated while using an Instron 8801 testing machine, in which the speed was 1 mm⋅s^−1^ for a 0–1 displacement and 5 mm⋅s^−1^, and the measurements were obtained at room temperature. The load force-elongation data were recorded by tensile tests that were carried out on the specimens with a gauge length of 12.5 mm. The samples after the tensile tests were cut parallel to the extrusion direction (X-Y) from the central parts of the Cu plates by an electro discharge machining (EDM) device. The classic dumbbell-shaped samples had a 17 × 3.5 × 1.2 mm dimensions and they were used for the measurements. The fractured samples were observed by stereoscopic and scanning electron microscopy. After each pass, the Vickers micro-hardness measurements were conducted on vertical-longitudinal sample sections while using a 200 G load.

## 3. Results and Discussions

### 3.1. Deformation Behavior

**Figure 3:**
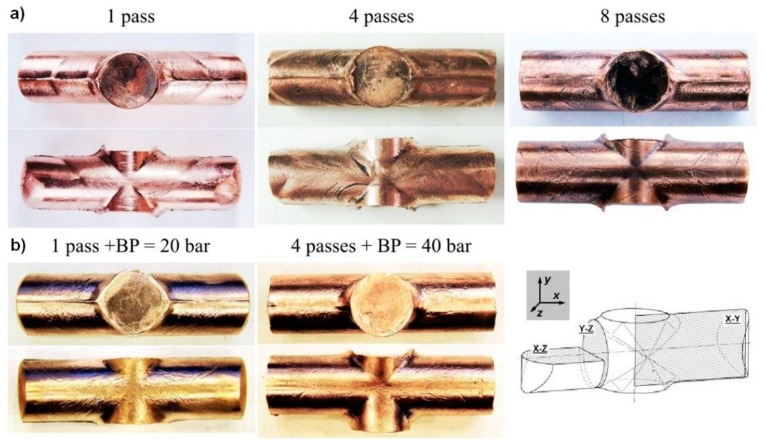
Of samples after different variants of the CCE process, (**a**) without and (**b**) with back pressure (BP).

Figure 3 shows images of the CCE samples that were processed with one to eight passes and obtained with and without back pressure (BP). The samples receiving one pass had outer surfaces with numerous slip bands that were caused by strong shear deformation. These bands form a characteristic pattern that resembles the letter “X”, which symmetrically extends along the axis of the sample. The pistons unevenly approached the horizontal axis of the outlet channel during extrusion, which disrupted the distortion symmetry and displaced the bands in one (lower) direction.

In the present case, these displaced bands are only visible on one side of the sample, the upper side. In the central part of the sample, an hourglass-shaped zone was formed with a slight degree of deformation, which is the remnant of the original ends of the sample. The newly formed sample fronts, which are also remnants of the original material, have a characteristic rounding in the X-Z plane. This finding indicates that there is considerable tensile stress that is concentrated in this region. This accumulated tensile stress causes increased the material pressure on the walls of the die channel, which increases the friction resistance and inhibition of the extruded material. However, in the vertical plane (X-Y), no such behavior was observed, which might indicate that the zone of concentrated stresses and the accompanying deformations is below the external surface of the sample.

Similarly, the authors of papers that were devoted to ECAP, regardless of the material in which they carried out the research, indicate the formation of a sample in the bottom zone (i.e., the X-Y plane along the symmetry axis of the output channel in this work) of a strongly deformed layer. The formation of this sample was associated with stresses occurring in this region and, especially, with the friction at the sample-matrix border [37,38,39,40]. At the same time, the top surface of the sample, due to lower stresses, was characterized by a much lower quality and presenting numerous furrows and even faults caused by the shearing of the individual material batches [22].

In the second and subsequent passages, the die with the sample was rotated by 90° (relative to the Z axis) and re-pressed in the opposite direction. Thus, the pistons started to press on the unloaded ends. The samples were repeatedly squeezed, which indicated that the distribution of strain on their surfaces became more uniform. Deformation is propagated in the entire volume of the sample, except in zones in the immediate vicinity of the frontal surface. The resulting patterns resemble triangular loops or they remain parallel to them, which is similar to the results that were described by Chou et al. [19]. As the number of passes increases, the size of the loop increases (Figure 3, four and eight passes). At the same time, furrows and small creases appeared. These furrows and creases may be indicative of an insufficient hydrostatic pressure in the materials, and such a weak pressure on the die wall causes these defects. Regardless of the processing changes, the CCE extruded samples do not contain cracks or other defects related to the loss of material integrity.

While assuming that the increase in the deformation homogeneity was due to introducing back pressure [21,22], the experiment was repeated using a second set of pistons, as described in [25]. As shown in Figure 3b, the introduction of a BP = 20 bar resulted in a marked improvement in the shape of the sample and a reduction in the number of defects that were created on the sample surface. Further increasing the BP and performing up to four passes practically leads to the disappearance of these effects, and the shape of the cylinder was preserved with negligible deformation to the shape. The application of a BP to the ECAP process eliminated the billet defects and produced work pieces that were nearly free of defects and highly suitable for commercialization, as reported by Mogucheva et al. [41].

### 3.2. Macro- and Microstructure

**Figure 4:**
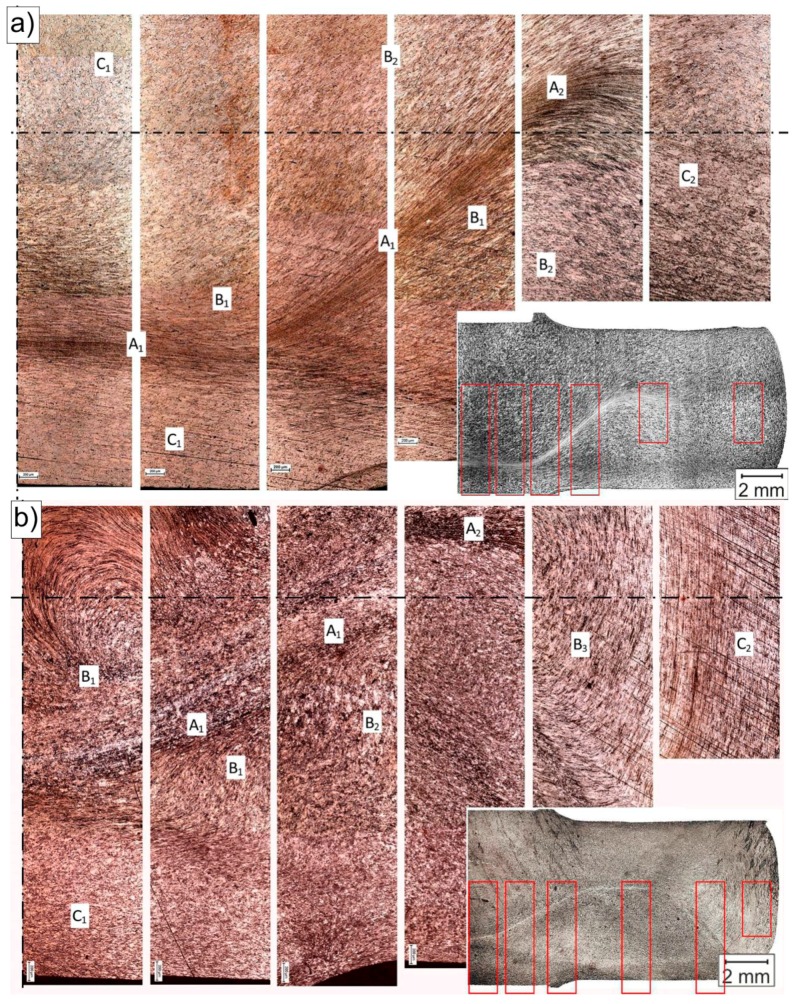
Of CCE specimens (light optical microscopy (LOM)): (**a**) one pass, (**b**) four passes (without BP).

Figure 4 shows the macrostructure images of the copper samples after being extruded by CCE one- and four-times. The resulting patterns show significant resemblance to those that were obtained from the clay modeling (Figure 2b). However, a significantly deformed zone was created (_−_/^−^) with a kinked shape due to the imperfections of the used die (i.e., inaccurate synchronization of the piston movement and stoppage) (Figure 4a). From a technological point of view, this fact has no significant impact on the final effect of the process. In addition, it can increase the homogeneity of the deformation by dispersing the deformation, as seen in Figure 4b.

The uniformity of the strain distribution in the deformation plane ensures the homogeneity of the deformation structure and the mechanical properties of the material [5,21]. However, non-uniform triaxial compression and shear deformation usually occur during CCE [17,23,24]. As a result, the deformation microstructure of the billet is essentially macroscopically inhomogeneous. An analysis of the observed structures (LOMs) allows for the microstructures to be divided into three types, depending on the place of occurrence and the method of deformation.

**Figure 5:**
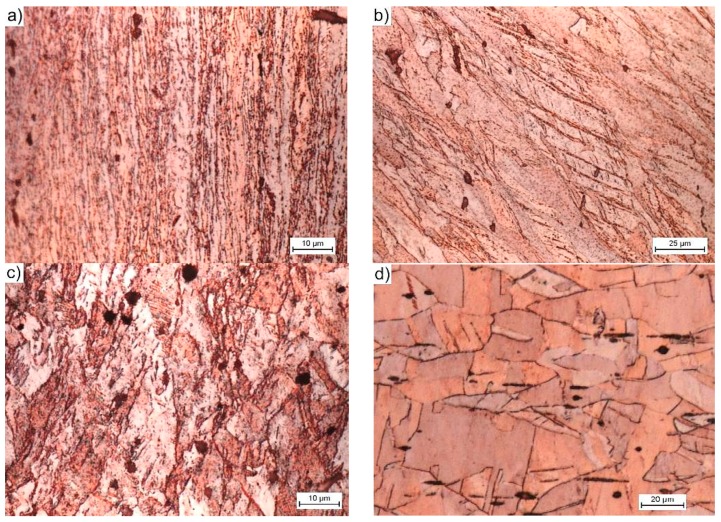
microstructure (LOM) processed by one pass of CCE. The structures are representative for the areas: (**a**) the central zone of strong compressive strains (A1 type band structure), (**b**) strongly deformed transition areas (type B1 band structure), (**c**) deformed as a result of shearing (grain structure deformed type B2), and (**d**) not deformed in the shear process (grain structure with small deformation, type C1 or C2, respectively).

In the centrally located zone A, which lies at the intersection of both channels, and as a result of the very strong compressive stresses and triaxial state of stress, the resulting structure was characterized by very strong deformations and a significant deformation of the structure. After a single pass, the structure forms strongly elongated bands, in which the grain boundaries (GBs) disappear in the classical sense of the term (Figure 5a). Within the structure, the strain was ε = 3–5.5, which represents approximately 20% of the entire sample volume, as estimated by the FEM simulation [23]. The width of a single band varies from tenths of parts to just a few micrometers, depending on the site of the study.

In zone B, there are still large deformations that extend on both sides of zone A. These deformations occurred as a result of the material that passes through the planes of the cutting stress, are inclined at an angle of 45° from both axes of the sample, and intersect at the midpoint of the cross channel of the matrix. Within the structure, we still deal with the bandwidth, but the grainy structure dominates. The elongation of a single grain was often up to 200 μm and the width was less than 15 μm. Depending on the deformation (on average for this zone is 1–1.1), the occurrence of shear bands (B1—Figure 5b) or effects related to their size distribution (B2—Figure 5c) was observed in the grain volume. The gradually decreasing role of the compressive stresses along with the distance from the central point and the increasing role of the shear stresses acting along the diagonal lines of the matrix channels caused this behavior. The grain shape reflects the strain path that was imposed by the shape of the channels, which is similar to the results of [18,38].

However, in zone C, the deformation of the granular structure was relatively small. Zone C consists of two cone-shaped areas that are directly located under the pistons (C1) and two similar ones that are located at the new opposite ends of the sample (C2). In these areas, the material had a structure that was similar to the initial state structure (Figure 5d). This behavior was related to the fact that it did not pass through the cutting stress plane (zone B) or exist in the zone of the hydrostatic compressive stresses (zone A).

**Figure 6:**
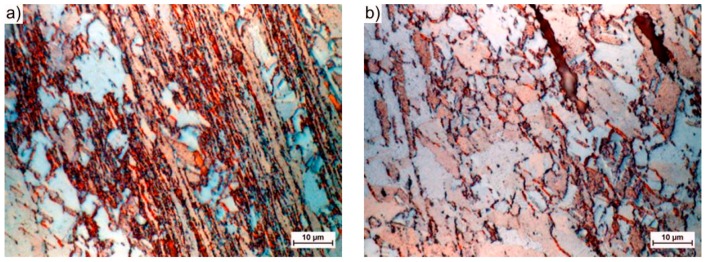

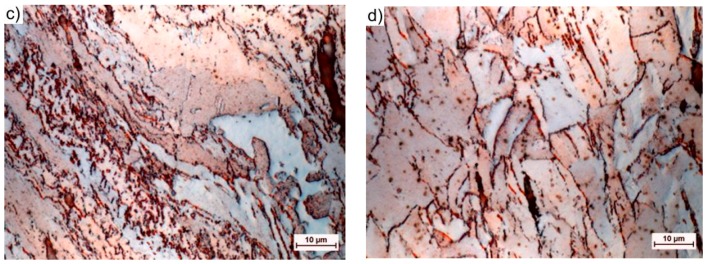
Microstructure (LOM) processed by four passes of CCE; (**a**–**d**) – description, as in Figure 5.

As the number of passes increases, the degree of structural deformation increases (Figure 4b). After four passes, the zone with high strains (A) extending centrally in the squeezing direction is still clearly visible. In addition to the abovementioned banding and blurring of the GB contours, new recrystallized grains appear in this structure (Figure 6a). The grain size, which depends on the energy stored, varies from a few to a dozen or so micrometers. The grains in zone B arrange themselves into strands due to the influence of the multiple passes through the plane of the cutting stresses, which propagate towards the outer surface of the sample at an angle of 45° to the axis and the center of the sample. The range and homogeneity of this zone increases with increasing numbers of passes. The resulting structure is typically granular (Figure 6b). In addition to the small 1–2 μm grains, there are larger grains with sizes of even several dozen micrometers. The shape resembles deformed primary grains with poorly marked substructures (Figure 6c). This finding suggests that, in this area, the structure was primarily rebuilt by dynamic recrystallization [42]. Additionally, in the remaining volume of the material, i.e., the zones of blocked stresses occurring directly under the pistons (C1) and on the tops of the sample (C2), phenomena leading to the fragmentation of the structure of the extruded material were observed. The fragmentation intensity was not as high in zone C, as it was in zones A and B, but the grain size within zone C more than doubled (Figure 6d). This process indicates that the entire volume of the extruded material undergoes plastic working with the appropriate number of passes.

These results are consistent with those that were presented by Chou et al. [19,20]. After extrusion, the specimen was deformed to form a symmetrical pattern, whose lines converged at the central point of the sample and were inclined toward the axis at an angle of 45°, as abovementioned. We can compare the results while assuming that the shape of the ECAP extruded sample by variant A corresponds to approximately ¼ of the shape that was obtained from CCE [1].

It was observed that after five passages of the ECAP, strong filamentary microstructure created with an increasing number of passes [3]. It was confirmed by the TEM investigation that many metals with a UFG size (under 1 mm) formed as a result of ECAP processes. Grains elongated along the shear direction, and the formation of elongated LABs was observed in the ECAP-ed aluminum alloy when ε ≈ 2 [41]. However, the increased strains experienced in the subsequent passes led the lamellar structure to breakdown and transform into a fine-grained structure.

Quite recently, Estrin et al. [43] presented an article dealing with archimats—materials whose properties are determined by their structure and chemical composition. Designing the internal architecture of the material is aimed at improving their mechanical and physical properties. Creating specific patterns in the structure is a characteristic feature of SPD techniques. Combining the possibility of obtaining a UFG structure with its controlled self-organization (in the case of multiphase materials) and extremely fast mass transfer leading to the creation of new phases opens new perspectives regarding the use of these techniques. Additionally, Cao et al. [44] present a similar approach while using the HPT technique.

While considering the above, the structures that were obtained by CCE processing and presented in Figure 2b and Figure 4, Figure 5 and Figure 6, fit perfectly into this current. Combining fine-grained structures that are responsible for high strength of the material with appropriately placed zones with a coarse-grained structure that usually improves their low plasticity is a kind of novelty, which the authors will have to face in the future.

#### 3.2.1. EBSD Analyze

**Figure 7:**
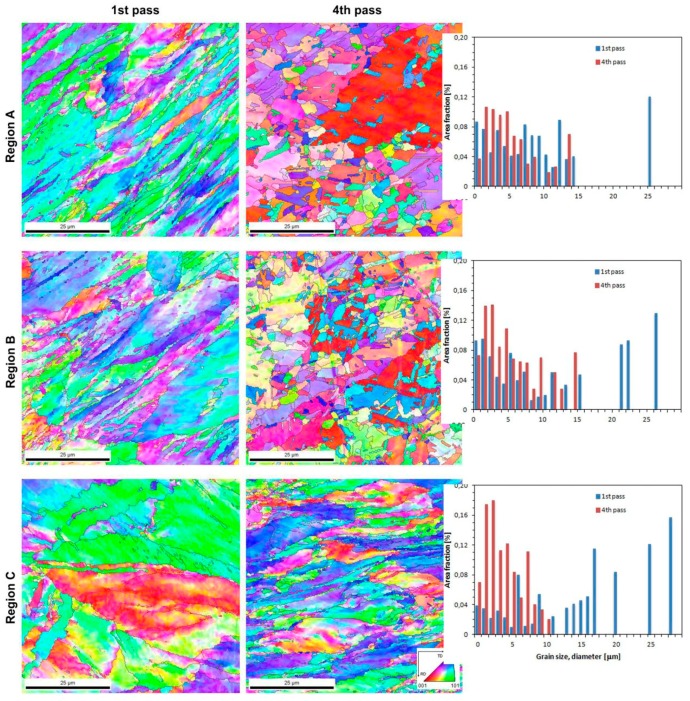
Inverse pole figure maps and grain size distributions for different regions of one and four passes of CCE.

Figure 7 shows the EBSD (inverse pole figure) maps and grain size distributions (d_eq_) of copper receiving one and four CCE passes. The analysis was carried out for three representative areas (A, B, and C). It can be seen that, after one pass, there are strongly elongated grains in zones A and B, which have a length-to-width ratio of at least 1:4. At the same time, the presence of fine grains of sizes less than 4 μm was also observed, and these grains were preferentially located near the GB or strain bands. The presence of these fine grains might indicate the dynamic reconstruction of the material structure. However, the grains in zone A are generally smaller and more elongated than the grains in the other zones and are arranged in characteristic bands; the structure of zone A is also more uniform than that of the other zones.

In zone A and B, the grain sizes range from 2 to 8 μm, and the shape factor value exceeds 0.4. In addition, zone A has been observed to contain grain bands of significantly larger sizes that are parallel to the extrusion direction. The FEM simulation that was performed for the Fe-Al alloy extruded by CCE shows that the alloy can be locally heated, even up to 180 °C in the central zone.

Hodowany et al. [45] estimated the temperature increase of a copper billet during ECAP. Assuming a typical yield stress of 350 MPa (representative of UFG copper) and ε ≈ 1, the temperature increased by approximately 64 °C for ECAP speeds of 1 mm/s. Wang et al. [46] investigated the effect of the temperature on the structure and properties of copper that was extruded by the ECAP method, and a similar type of structure was observed for samples that were statically annealed at 150 °C for 10 min. Wang et al. indicated that, after the recrystallization of the material that took place at 120 °C, some grains tended to grow, forming characteristic twins. Thus, in the present research, due to the energy accumulated in the material, the material undergoes spontaneous dynamic recrystallization and, even locally, grain growth, which might lead to secondary recrystallization.

Depending on the deformation and the dominant type of stress, the material in zone B exhibits a variety of structures, which is similar to what was observed in the LOM photographs. Where the cutting stress was very intense and the deformation path frequently changed, a grain size of only 2 μm was created. In other regions, large grains have been preserved, but they are cut and contain inclusions or whole bands of new small grains. At large strains, a large number of dislocation boundaries, which change their misorientation into high-angle GB, and a UFG structure are formed [47]. Vinogradov and Estrin [48] suggest that, during SPD, small deformations and grain refinement already occurs. However, the increase in the deformation (ε ≈ 1) triggers a competitive grain growth mechanism. Thus, the final result depends on the balance between the factors that favor these mechanisms.

The tests that were carried out in zone C, i.e., in the cone zones near the surface of the piston or on the front of the sample, indicate that, although they did not cross the planes of the shearing stresses, zone C was deformed (Figure 7 Region C). Zone C is dominated by relatively large, several-micron long grains with elongated shapes. However, at the long grain borders, small aggregations (1–5 μm) form in large clusters that look like necklace recrystallization [49]. This proves that the CCE-extruded material is able to achieve satisfactory mechanical properties from a construction point of view with the right number of passes.

It is known that the grain refinement that is achieved during SPD is basically controlled by two factors: process parameters, such as the strain, strain rate, temperature, and deformation path, or material parameters, such as the initial grain size and stacking fault energy (SFE). Tao and Lu [50] reported that increasing the strain rate and/or decreasing the temperature leads to the dominating refinement mechanism changing from slip by dislocations to twinning. The deformation in high SFE materials is subjected by slip, even at relatively highs strain rates and/or at low temperatures. For low SFE metals, twinning dominates structural refinement [51]. The deformation twins are much more difficult to form in FCC metals than dislocation slip [41], especially in FCC metals with a higher SFE, such as copper, nickel, and aluminum [52]. In addition, the crystallographic orientations and grain geometry also have significant influence on the material’s deformation mechanism [53].

Xue et al. [54] proposed a mechanism explaining the grain refinement of Cu that occurs during the first pass of ECAP. This model explains that the microstructural changes usually consist of four stages—dislocation generation, dislocation cell construction, gliding along the main slip planes and elongated laminar structure (ELS) formation, and the possible formation of a second set of ELSs and/or micro-shear bands (MSBs).

In contrast, Sakai et al. [47] suggested that the MSBs that formed during the next passes crossed each other to form equiaxed structures at intersections and later formed along the bands (chessboard structure), which is similar to the result that is presented in [41]. Further processing increases the density of MSBs and resulted in more UFG equiaxed structures forming. The grain size achieved by SPD techniques is determined by the dynamic equilibrium between the generation and recovery of dislocations and grain refinement or growth [55].

The CCE process involves repeatedly pressing a material in opposite directions. There are only a few articles in the literature that show that grain growth is a consequence of large deformations or strain reversals that occur during SPD processes. Bagherpour et al. studied the role of strain reversal in softening (Bauschinger effect) and the resulting structure [56] They extruded pure Cu samples (SSE at ECAP in route C) process, and the resulting samples contained evidence of shear strain reversal. Bagherpour et al. stated that the grain subdivision is the main mechanism driving the forward shear. By reversing the shear, the dislocation fluxes are reversed, which reduces the number of stored dislocations and retards the formation of HAGBs.

#### 3.2.2. Microhardness

**Figure 8:**
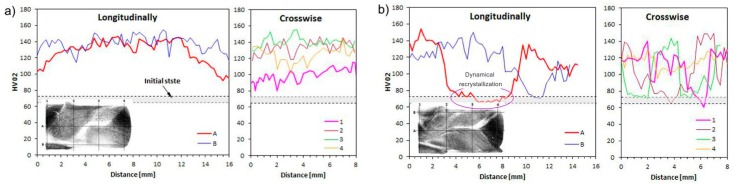
Evolution during CCE process after: (**a**) one pass and (**b**) four passes. A, B—longitudinal and 1–4—transverse paths of microhardness measurement.

Figure 8 illustrates the relationship between the number of deformation cycles and the microhardness of CCE-processed copper. The measured values are closely related to the observations of the microstructure and, as such, the measured values and observations should be considered together.

As seen in Figure 8a, after the first pass, the material strengthened relatively uniformly, both in the interior and in the superficial area. The microhardness increased from 69 ± 4 HV0.2 to approximately 130 HV0.2, regardless of whether the measurements were made in zone A or B. The increase in the microhardness measured in zone C, due to the low degree of deformation, was approximately 50% smaller than that in the other zones.

An increase in the deformation after four passes does not lead to an increase in the microhardness but instead leads to a decrease in the microhardness (Figure 8b). This phenomenon was observed everywhere in locations where the measurements hit the zone with the most deformations, i.e., the lamellar zone A. Within this zone, the copper microhardness dropped to the baseline. It can be assumed that a significant accumulation of energy in the volume of strongly deformed material caused this phenomenon. As presented in Section 3.2.3, this energy turns out to be so large that the temperature of the beginning of thermo-structural transformations is lowered and spontaneous initiation of dynamic recrystallization occurs. In zone B, the microhardness remained at a similar or slightly lower level than that achieved after being extruded once by CCE. However, in zone C, the microhardness increased to 120 HV0.2 as a result of the gradual propagation of deformations.

This situation might indicate two things. First, even during the first pass, the entire volume of the material is deformed, but to a different deformation degree. Second, although the created hydrostatic compressive stresses are responsible for the formation of the strong deformation zone (A), the shear stresses are responsible for the favorable transformations that are associated with the fragmentation of the coarse-grained structure of the extruded material.

The increase in the material hardness due to its deformation is a well-known fact. In the SPD processes, the hardness is usually observed as almost double after the first pass and then increases further, but the hardness gradually decreases after subsequent passes [21,41,57,58]. However, this study showed a different result, as the hardness declined. As mentioned earlier, this behavior was associated with progressive secondary recrystallization and the disappearance of the beneficial effects of structural fragmentation. Thus, it can be concluded that we are able to introduce and accumulate a large portion of energy in the material by using CCE extrusion, and not only dynamic recrystallization, but also secondary recrystallization, will be initiated.

A similar behavior was observed by Lachhab et al. [59] for an ECAP-ed Fe-48Ni (wt. %) alloy, where the microhardness increased by nearly two times after the second passes. The Hall–Petch effect and dislocation density were evaluated to be most the responsible for strengthening the material.

#### 3.2.3. DSC

**Figure 9:**
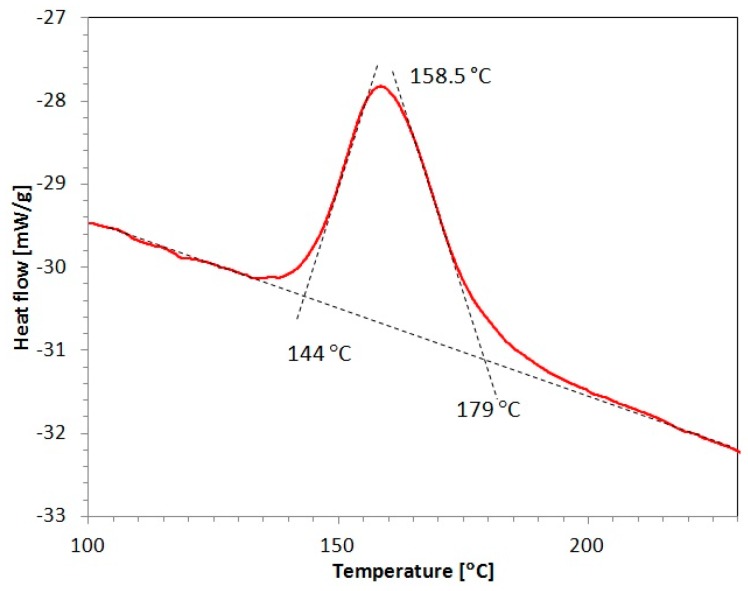
Of differential scanning calorimetry differential scanning calorimetry (DSC) of copper after four passes CCE.

DSC was performed to evaluate the stored material strain energy for a copper sample extruded four times by CCE, and Figure 9 shows the results. The DSC curve shows one exothermic peak at a maximum temperature of 158.5 °C, which is unambiguously associated with the energy that is released during a conventional static crystallization process [60]. The amount of energy calculated from the area under the curve was estimated to be 53 ± 2.1 J/mol (0.84 ± 0.03 J/g), which is close to the values that were obtained by Cao et al. [61] for severely deformed copper by the equal channel angular pressing technique. After one ECAP pass, Cao et al. observed an exothermic peak at 215 °C with a 0.56 J/g stored energy and another peak at 163 °C with a 0.95 J/g for the samples analyzed after 4–12 passes. Higuera-Cobos and Cabrera observed slightly higher values in similar conditions [62].

Marković et al. [63] investigated the thermal stability of cold rolled Cu-12.7Au% wt. with 60% work-up and observed the occurrence of three exothermic peaks from the DTA curve. The first peak occurred at a temperature of 210 °C and it was associated with the annihilation of point defects. The other peaks occurred in the temperature range from 210 °C to 405 °C and at 490 °C, and their appearance was not directly related to the plastic working.

The activation energy (Q) of recrystallization substantially decreases with increasing the number of passes, which is consistent with other reports [62,64]. For example, the stored energy obtained for pure Cu extruded twice by ECAP and determined by thermodynamic calculations (0.43 J/g) was lower than that of the sample that was extruded 16 times by ECAP (0.66 J/g). Thus, the Q values of the Cu samples extruded two times and 16 times by ECAP were 94 and 72 kJ/mol, respectively [65].

The obtained results confirm the earlier assumptions regarding the possibility that not only dynamic recrystallization, but also secondary recrystallization occurs during treatments while using the CCE technique. On the one hand, the accumulated energy significantly lowers the temperature, in which recrystallization starts, and, on the other hand, a strong deformation can lead to the accumulated energy exceeding in a very significant way. Thus, as observed in this work, a recrystallized and fine-crystalline structure forms, and the individual grains selectively grow.

#### 3.2.4. Resistivity

The resistivity (specific resistance) determines the conductive properties of a conductor and determines the resistance of a conductor with a length of one meter and a cross-section area of 1 m^2^. The resistivity is a constant value that characterizes a given material; for pure copper, (99.999% Cu) the resistivity is 1.72 × 10^−8^ Ω⋅m [36], but, in this case (99.9% Cu), the resistivity was an order of magnitude larger. As shown in Table 1, a deformation once caused the resistivity to decrease by almost four-fold. Further increases in the deformation (4x CCE) and additional heating at 200 °C by 10 min. both caused an increase in the resistivity. Such a behavior indicates that the dislocations that arise in the material at a certain level may favor the conductivity of the electric current, which lowers the resistance of the material. Likewise, the grain size might decrease to the nanometer range, but in this study, this effect was not very observed.

The abovementioned results are in contrast with the literature data, where grain refinement by mtECAP [66] caused a 7% increase in copper’s resistivity, which is similar to the results that were shown by [29,62] and was explained by the electron scattering caused by the structural defects. The increase in the electrical resistivity that is caused by grain refinement is not significant and, for pure copper, is on average 2–4%.

Qi et al. observed a similar behavior to that described in this article [28]. After achieving an SPD by cyclic extrusion of approximately 1000% at 400 °C, the conductivity of the AA6061-T651 alloy increased by 10% IACS, which means that the resistivity decreased by approximately 20%. Furthermore, aging results in a slight decrease in the conductivity, which still remained higher than in the original state. It was shown that the fracture and re-distribution of macro-precipitates due to severe shear and few dislocations are beneficial to the conductivity. In contrast, Murashkin et al. [67] investigated an Al-Mg-Zr alloy that was deformed by ECAP-C (Conform) and, subsequently, by cold drawing, and Higuera-Cobos and Cabrera [62], found that the conductivity of pure copper only very slightly changed due to SPD processing.

As abovementioned, the electrical properties of an SPD-ed material are not obvious, and the resistivity or electrical conductivity is not a simple function of the degree of material fragmentation or the density of accumulated dislocations.

#### 3.2.5. Static Tensile Test

**Figure 10:**
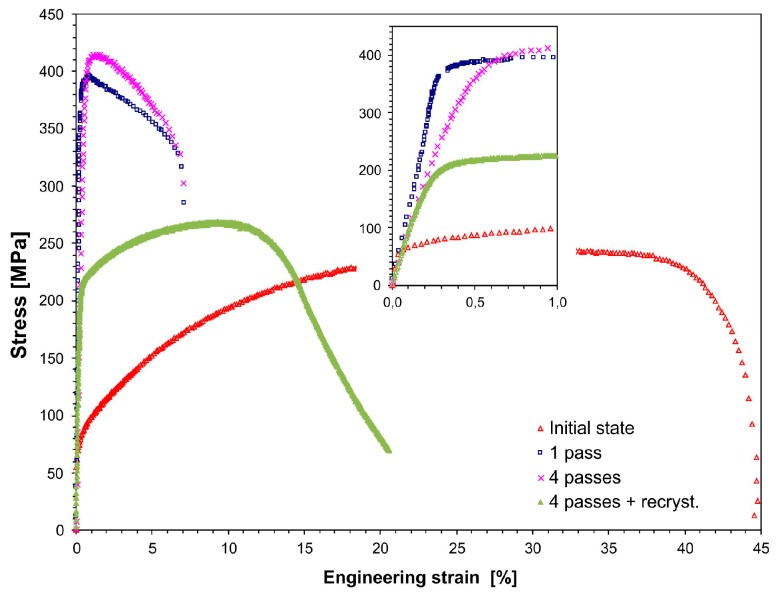
Stress-strain curves for as-received and CCE-processed copper.

Table 2 lists the tensile properties for the copper specimen that underwent 1–4 passes of the CCE process. Additionally, the results of the samples subjected to four passed and to static recrystallization at 200 °C for 10 min. are also shown. Figure 10 shows the representative engineering stress-strain curves for each condition. The values of the ultimate strength (UTS), yield strength (YS), total elongation (A), and reduction in area (RA) were obtained from the engineering stress-strain curves and the tested samples.

The yield and ultimate tensile strength exhibited a pronounced increase from 75 and 241 MPa to 390 and 396 MPa, respectively, after one pass of the CCE process. These results, combined with a nearly four-fold decrease in the elongation to ~12%, indicate the strong deformation strengthening of the material and deformation of the primary copper structure. A further increase in the deformation only leads to a small increase in these parameters up to 403 and 415 MPa. This corresponds to a nearly two-fold increase from the initial state. On the other hand, these parameters decreased to 218 and 269 MPa with a simultaneous increase in the plasticity to 20% after additional recrystallization annealing.

The process of homogenizing in the subsequent cycles of extruding the microstructure created small equiaxed grains, which is accompanied by the homogenization of the deformation process and the mechanical properties of the resulting material. It can be assumed that proof stress saturation and UTS could occur, as was observed, for instance, for the copper specimen that was processed by ECAP [68,69]. The results that were obtained should also be associated with the fact that the material was greatly strengthened, and a mixture of sub-micron and micron sized grains were produced. The former gives the material high strength, while the latter is responsible for the relatively good ductility, as shown in [63]. The distinct differences in the mechanical strength were caused by the grain boundaries and dislocation strengthening mechanisms. Similar results were reported for the tensile tests that were performed on the sample that was extruded by CCE once [66], where the UTS was approximately 400 MPa and the total elongation was approximately 13% after extruding six times by ECAP with two turns (route B_C_); similar results were also described in [70] for samples receiving 10 passes of ECAP (B_C_). Additionally, after ARB was performed four times [24], the UTS values were similar, while the average grain size was approximately 200 nm, which is significantly lower than the grain size that was obtained in the present study. A reason for the yield strength increase is that the finer boundary spacing was considered according to the Hall–Petch relationship.

**Figure 11:**
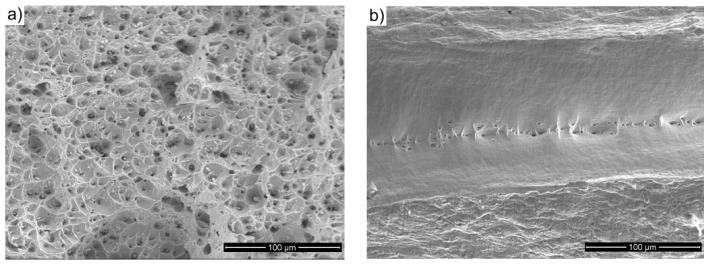
Of Cu processed by CCE: (**a**)—one pass, (**b**)—four passes with recrystallization.

The abovementioned results are reflected in the fractographic studies that were performed on the tensile fractures of the samples. In the initial state, after copper was annealed at 500 °C for one hour, a typical ductile transcrystalline technique characterized the copper samples. As a result, there was a severe narrowing in the area of rupture (RA = 89%), which formed a crater whose edges were parallel to the external surfaces of the sample, and the surfaces were inclined at an angle of 45° to the stretching direction. These surfaces were smooth, and only characteristic cavities were observed at the bottom of the crater. However, after the extrusion process, if the character of the breakthrough did not change the remaining plastic character, the shape changed instead. On the surface, we can see numerous conical wells with small spherical particles on the bottom of the wells. They were not torn apart regardless of the number of deformation cycles, which would indicate an increase in the brittleness of the material (Figure 11a). However, by increasing the deformation that was achieved in the subsequent passes, the surface of the breakthrough increases. At the same time, the surface becomes flatter; the surface was wavy after the first pass and, after the fourth pass, it was practically flat and perpendicular to the stretching direction. These morphological changes are accompanied by a gradual reduction in the depth of the abovementioned recesses and the elongation of the sample, which was also observed for severely deformed copper [65].

However, after four passes of CCE and an additional recrystallization annealing process, we observe that the properties return to those of the initial state. If the strength decreases, the plasticity increases. At the same time, the abovementioned crater reappears on the surface of the breakthrough (Figure 11b). This time, the crater is smaller, which corresponds to a larger reduction rate (92%), and its surface is flat and has negligible traces of plastic fracture. However, this finding does not indicate the emergence of a brittle breakthrough, but instead indicates significant fragmentation of the coarse grain structure and plastic working.

The fatigue fracture of the samples after ECAP strongly differs from the surface relief of the samples in the coarse-grained state and the deformation parameters, and other authors have reported similar copper behaviors that are associated with the plastic breakthrough that occurs with an increase in the deformation [62,66] or the reduction in the number of dimples and created cleavage planes, which indicated the existence of a mixed ductile-brittle fracture type, as has been demonstrated by the tensile ductility reduction [71].

**Figure 12:**
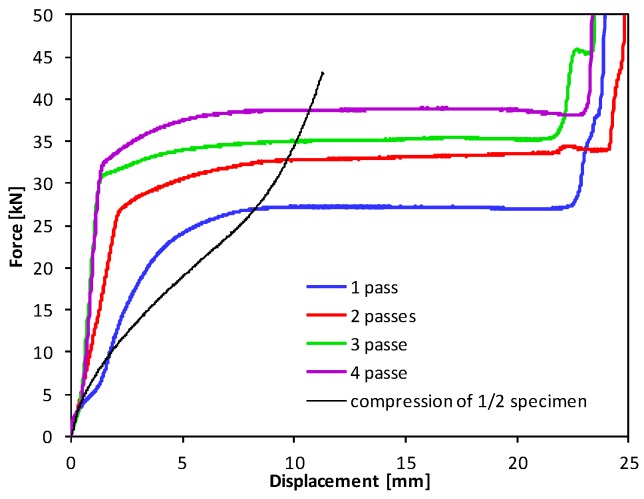
Forces during CCE process.

Extrusion tests were carried out using various parameters to verify the process of CCE and evaluate the strengthening that occurs during CCE, using the Instron 8801 testing machine. Figure 12 shows the force-displacement curves for the specimens receiving 1–4 passes. For comparison, the graph shows the compression curve of a copper sample with the same diameter, but half the height relative to the sample used for CCE. Extrusion can be divided into two stages. The first stage, in which the force increases very quickly, is associated with filling the central part of the matrix along the arched passage of the channel entrance into the channel exit (only for one passage) and forming the top of the reorganized sample.

The second stage corresponds to the plastic flow of the material with a practically constant extrusion force. The extrusion process ends when the pistons reach the stop surfaces due to the rapid increase in strength. By applying subsequent passes, the material strengthens, the curves obtained during the first stage become more vertical, and a shorter transition period is observed during the second stage. While the force needed to crush the sample after the first pass was approximately 25 kN, this force increased to 38 kN after the fourth pass. When comparing these results to the free compression curve of an analogous copper sample (shown by the black line in Figure 12), we see that the closure of the workpiece with a limited volume of the matrix only increases the resistance of its deformation in the initial stage. Assuming that the channel lengths were equal, then a 20 mm piston displacement (2.5 times the diameter of the channel) force is needed to compress the sample, which exceeds the force of extrusion. The elongation of the channels and the sample itself will not increase the force that is necessary to achieve this, as opposed to processes that produce materials with unlimited expansion. However, increasing the dimensions of the matrix creates many technological problems.

## 4. Conclusions

This article presents the feasibility of using the CCE process for refining the grain structure in copper with the objective of improving the copper characteristics. Billets were successfully processed by four CCE passes with and without applying a back pressure. The following conclusions are drawn from the results obtained:

1. The CCE process has been shown to be an effective method that can produce flaw-free billets. This promising technique requires relatively little pressure. In addition, the introduction of a BP improves the uniformity of the billets and allows for their cohesion.
2. Processing a material by CCE, due to the large unit deformation values, results in the formation of a lamellar structure along the extruded axis, resulting from triaxial compression (zone A) and the fine-grained structure that results from the impact of the shear stress in the remaining volume of the material (zone B).
3. The severely deformed material exhibited the initiation of dynamic recrystallization, which results in the formation of 0.5 to 2 μm grains after a single pass and 2- to 8-μm grains after four passes.
4. An increase in the copper microhardness from 70 to 130 HV02 after one CCE pass indicates that all of the material undergoes deformation with considerable diversity. While the microhardness decrease (even to 70 HV02) and the increase in the number of passes may result in progressive recrystallization. This agrees with the exothermic peak that was observed during the DSC testing the CCE-treated copper billets. The temperature in which this exothermic peak was observed (158.5 °C) and the amount of accumulated energy (53 ± 2.1 J/mol) suggest that in such a deformed material, secondary recrystallization, and selective grain growth may also occur.
5. The grain refinement caused an improvement in the YS and UTS of approximately 50%, and the elongation to failure decreased to a satisfactory value of 9.7%.
6. The resistivity of the once deformed copper significantly decreases, while further processing the copper specimens causes the resistivity to increase.

## Figures and Tables

**Table 1:** Resistivity (10^−8^ Ω⋅m) of copper after different CCE process.

Initial (Recrystallized)	1x CCE	4x CCE	4x CCE + Recrystallization
19.8	5.7	7.9	11.9

**Table 2:** Tensile properties of coarse grained and CCE extruded Cu.

Number of Passes	YS (MPa)	UTS (MPa)	A (%)	RA (%)
annealed	75	241	42.4	89
1	390	396	11.8	49
4	403	415	9.7	44
4 + recrystallization	218	269	20.8	92
